# High Numbers of CD163+ Tumor-Associated Macrophages Predict Poor Prognosis in HER2+ Breast Cancer

**DOI:** 10.3390/cancers16030634

**Published:** 2024-02-01

**Authors:** Minna M. Jääskeläinen, Ritva Tumelius, Kirsi Hämäläinen, Kirsi Rilla, Sanna Oikari, Aino Rönkä, Tuomas Selander, Arto Mannermaa, Satu Tiainen, Päivi Auvinen

**Affiliations:** 1 Cancer Center, Kuopio University Hospital, Wellbeing Services County of North Savo, 70029 Kuopio, Finland; 2 Institute of Clinical Medicine, University of Eastern Finland, 70211 Kuopio, Finland; 3 Kuopio Center for Gene and Cell Therapy, 70210 Kuopio, Finland; 4 Institute of Clinical Medicine, Clinical Pathology and Forensic Medicine, University of Eastern Finland, 70211 Kuopio, Finland; 5 Imaging Center, Clinical Pathology, Kuopio University Hospital, Wellbeing Services County of North Savo, 70029 Kuopio, Finland; 6 Biocenter Kuopio and Cancer Center of Eastern Finland, University of Eastern Finland, 70211 Kuopio, Finland; 7 Institute of Biomedicine, University of Eastern Finland, 70211 Kuopio, Finland; 8 Science Services Center, Kuopio University Hospital, Wellbeing Services County of North Savo, 70029 Kuopio, Finland; 9 Biobank of Eastern Finland, Kuopio University Hospital, Wellbeing Services County of North Savo, 700029 Kuopio, Finland

**Keywords:** tumor-associated macrophage, HER2, CD163, CD68, breast cancer, trastuzumab

## Abstract

**Simple Summary:**

While tumor-associated macrophages (TAMs) are known to be associated with a poor prognosis in breast cancer (BC) in general, how they influence different BC subtypes has remained poorly studied. Here, we investigated the prognostic value of M2-like TAMs (CD163+) that mediate several pro-tumoral functions, and all TAMs (CD68+) in a patient cohort of 278 non-metastatic BC cases, half of which were HER2+. Our results indicate that M2-like TAMs were associated with a poor outcome in HER2+ BC, in which a high CD163+ TAM count was independently associated with an inferior outcome regardless of hormone receptor status and the use of adjuvant trastuzumab. In summary, our results suggest that the prognostic value of M2-like TAMs is especially significant in HER2+ BC; these patients might benefit from combination treatments that include therapies targeting macrophage function.

**Abstract:**

Tumor-associated macrophages (TAMs) are associated with a poor outcome in breast cancer (BC), but their prognostic value in different BC subtypes has remained somewhat unclear. Here, we investigated the prognostic value of M2-like TAMs (CD163+) and all TAMs (CD68+) in a patient cohort of 278 non-metastatic BC patients, half of whom were HER2+ (*n* = 139). The survival endpoints investigated were overall survival (OS), breast cancer-specific survival (BCSS) and disease-free survival (DFS). In the whole patient cohort (*n* = 278), a high CD163+ TAM count and a high CD68+ TAM count were associated with a worse outcome (*p* ≤ 0.023). In HER2+ BC, a high CD163+ TAM count was an independent factor for a poor prognosis across all the investigated survival endpoints (*p* < 0.001). The prognostic effect was evident in both the HER2+/hormone receptor-positive (*p* < 0.001) and HER2+/hormone receptor-negative (*p* ≤ 0.012) subgroups and regardless of the provision of adjuvant trastuzumab (*p* ≤ 0.002). In HER2-negative BC, the CD163+ TAM count was not significantly associated with survival. These results suggest that a high CD163+ TAM count predicts an inferior outcome, especially in HER2+ BC patients, and as adjuvant trastuzumab did not overcome the poor prognostic effect, combination treatments including therapies targeting the macrophage function could represent an effective therapeutic approach in HER2+ BC.

## 1. Introduction

Despite the improved prognosis in recent years, breast cancer is still the leading cause of cancer-related deaths in the global female population. The survival rates differ according to the breast cancer subtype. Hormone receptor-positive/HER2− (HR+/HER2−) breast cancer presents with the best 5-year survival rate of 94%, followed by HR+/HER2+ cases (91%) and hormone receptor-negative/HER2+ (HR−/HER2+) cases (85%), while triple-negative (HR−/HER2−) breast cancer has the worst 5-year survival rate of 77% [1]. While surgical treatment with or without radiotherapy has remained the backbone of the treatment of primary breast cancer, the administration of drug therapy has shifted more towards the neoadjuvant setting. Hormonal treatment remains the standard of care in early HR+/HER2− breast cancer either alone or combined with a CDK4/6-inhibitor for high-risk patients, while HER2- antibodies (trastuzumab with or without pertuzumab) combined with chemotherapy remain a cornerstone in the treatment of HER2+ breast cancer. The indications of the current immuno-oncological treatments, like pembrolizumab and atezolizumab, are restricted to triple-negative breast cancer [2].

The role of immune cells in the tumor microenvironment (TME) has been a topic of active research across various cancer types, including breast cancer. While the prognostic value of tumor-infiltrating lymphocytes (TILs) is now widely established [2,3,4], there is evidence that other types of immune cells, such as tumor-associated macrophages (TAMs), are also important in the progression of breast cancer [5,6]. In fact, TAMs are the most common immune cell type in the TME, and they play a significant role in disease development, progression and the response to therapy [6,7,8,9]. TAMs are cells which display properties of high plasticity and heterogeneity [9,10]. They can be divided into two main categories: classically activated M1-like macrophages (M1 TAMs) with anti-tumor properties and alternatively activated M2-like macrophages (M2 TAMs) with pro-tumoral effects [11]. CD68 is considered to be a pan-macrophage marker while CD163 is one of the commonly used markers for M2 TAMs [9,12]. Even though a high number of TAMs, especially the M2-like phenotype, is recognized to be associated with inferior outcomes in breast cancer [13,14,15,16], the prognostic effects of TAMs in different breast cancer subtypes have remained less well defined.

HER2+ breast cancer is an aggressive breast cancer subtype, even though anti-HER2-treatments, e.g., trastuzumab, have significantly improved its prognosis [2,17]. However, despite the improved treatments, some patients still suffer a relapse either locally or as a non-curable metastatic HER2+ breast cancer. In addition, de novo and acquired resistances to anti-HER2 treatments are a major challenge, especially in the metastatic setting [17,18].

Only a few investigators have examined the prognostic role of TAMs in HER2+ breast cancer [19,20,21,22]. We have previously reported that a high number of M2 TAMs was associated with poor overall survival (OS) among HER2+ breast cancer patients [19], whereas other researchers have reported no significant correlation between M2 TAM numbers and survival in this breast cancer subtype [20,21]. In summary, the prognostic role of TAMs has remained unresolved in HER2+ breast cancer. Interestingly, the diverse actions of trastuzumab include immunomodulatory effects [17,23]. For example, it is known that trastuzumab can induce an immune response against cancer cells, and thus one could speculate that the immunological conditions in the TME may influence the benefits of this kind of drug therapy. Indeed, a high number of TILs has been associated with a beneficial anti-HER2 antibody response [24]. Furthermore, TAMs have been postulated to exhibit contrasting effects, but the properties of these cells remain to be fully elucidated [24,25].

Considering the heterogeneous nature of breast cancer, it is clearly important to clarify the effects of immune cells in the TME in different breast cancer subtypes. In the present study, we focused on the prognostic role of M2 TAMs (CD163+) and all TAMs (CD68+) in early breast cancer according to HER2 status and hormone receptor status. Here, we report the 10-year OS, breast cancer-specific survival (BCSS) and disease-free survival (DFS) rates according to the number of M2 TAMs (CD163+) and all TAMs (CD68+) in a cohort of 278 non-metastatic breast cancer patients, every second of which was HER2+ (*n* = 139).

## 2. Materials and Methods

### 2.1. Patient Material and Study Design

The patient material of this study consisted of a real-world patient population operated upon in the Kuopio University Hospital between 2001 and 2008. First, all primary early HER2+ breast cancer cases (*n* = 163) operated upon in this period were collected. If only a core needle biopsy sample was available (patients receiving neoadjuvant therapy or inoperable tumors) or the representative tissue block was missing, the patients were excluded from this study (*n* = 24), leading to an HER2+ cohort of 139 patients. The HER2- cohort was composed of an equal number (*n* = 139) of cases matched with the patients’ age and operation time. Thus, the total patient cohort consisted of 278 patients. The collection of the study population has been described previously in greater detail [26]. Adjuvant treatments (chemotherapy, radiotherapy, hormonal therapy, trastuzumab) were given according to the clinician’s judgement adhering to the national guidelines. For this study, the survival follow-up was updated on 26 May 2020. In the updated clinical data, the median follow-up time of the whole patient cohort was 13.1 years (range 0.4–18.8 years). The prognostic value of CD163+ TAMs and CD68+ TAMs was analyzed, with the following clinical endpoints being investigated: OS, BCSS and DFS. The numbers of deaths and relapses in the different subgroups are presented in Supplementary Table S1. Ethical approval was issued by the Ethics Committee of the University of Eastern Finland (24 February 2009, 19//2009) and Kuopio University Hospital provided approval for the study (permit number 5654132). The study was conducted according to the Declaration of Helsinki. The REMARK criteria were followed when applicable [27].

### 2.2. Assessment of TAMs

CD163 positivity and CD68 positivity were considered indicators for M2 TAMs and all TAMs, respectively. The immunohistochemical staining and the assessment of TAMs have been described previously in greater detail [19]. Briefly, the microscopic evaluation of CD163+ and CD68+ TAMs was performed utilizing the hot spot method. The areas with the highest TAM densities (hot spots) were first scanned at a low magnification (×5–10 objective) of tumor material dissected during breast cancer surgery. Then, the numbers of TAMs were counted in at least four hot spots of each section (×40 objective) and averaged to represent the number of TAMs in the section. Examples of the CD163+ and CD68+ immunostainings are presented in the Supplementary Figure S1.

### 2.3. The Standard Histopathological Parameters

The standard histopathological parameters were assessed in routine diagnostics according to the WHO and the international guidelines of the time period [28,29,30,31] in the Department of Pathology, Kuopio University Hospital. Estrogen receptor (ER) and progesterone receptor (PR) statuses were assessed via an immunohistochemical assay of formalin-fixed, paraffin-embedded samples. The threshold for ER and PR positivity was >10% and cases were considered to be hormone receptor-positive if either ER or PR were positive. HER2 status was assessed using a chromogenic in situ hybridization (CISH) assay with the threshold for HER2 positivity being six or more gene copies per nucleus.

### 2.4. Statistical Methods

The statistical analyses were performed with IBM SPSS Statistics 27 (IBM Corporation, Armonk, NY, USA). The differences between investigated factors were calculated with the Mann–Whitney U-test and Chi-square test. Cox’s model and the Kaplan–Meier method were used for survival analyses and plotting the survival curves. The prognostic effects of CD163+ and CD68+ TAMs were evaluated as continuous variables and the hazard ratios (HRs) for each 10-unit increase in the TAM counts were determined. In the multivariate survival analyses and in plotting the survival curves, the TAM counts were dichotomized as low or high using optimal cut-off-points determined by using the receiver operating characteristic (ROC) curve with BCSS as the survival endpoint. The cut-off points for both CD163+ and CD68+ TAMs were determined separately for each investigated subgroup. The Cox multivariate survival analyses included the investigated immune cells (i.e., CD163+ TAMs and CD68+ TAMs) as well as the standard prognostic factors, i.e., tumor size (T2–4 vs. T1), nodal status (N1–3 vs. N0), grade (3 vs. 1–2), hormone receptor status, HER2 status and adjuvant trastuzumab for HER2+ cases. OS was determined as the time from diagnosis to the date of death from any cause or to the end of follow-up; BCSS was determined as the time from diagnosis to the date of breast cancer death or to the end of the follow-up; and DFS was determined as the date of diagnosis to disease recurrence (local or distant), death or the end of the follow-up. *p*-values <  0.05 were regarded as statistically significant.

## 3. Results

The clinicopathological characteristics of the patients and the prevalence of TAMs in different breast cancer subgroups are presented in Table 1. Half of the cases (*n* = 139) were HER2+, of which 82 (59%) were hormone receptor-positive (HR+) and the remaining 57 (41%) were hormone receptor-negative (HR−). The adjuvant treatments administered are presented in Table 1; 204 (73%) patients received chemotherapy, 248 (89%) radiation therapy and 174 (63%) hormonal therapy. Adjuvant trastuzumab therapy was given to 63 patients, i.e., 45% of HER2+ cases received this therapy. In comparison to the HER2- tumors, HER2+ tumors presented with more aggressive features, i.e., with a larger tumor size (*p* = 0.034), more advanced nodal status (*p* = 0.015) and higher grade (*p* < 0.001). In the HER2+ subgroup, HR− breast cancer was associated with a higher tumor grade when compared to HR+ cases (*p* = 0.024). The number of CD68+ TAMs was higher in HER2+ breast cancer in comparison to HER2- breast cancer cases (*p* < 0.001), whereas the CD163+ TAM count was not significantly associated with HER2 status (*p* = 0.132) (Table 1).

During the 13.1-year follow-up of the whole patient cohort, 91 (33%) patients suffered some form of relapse and 98 (35%) patients died, 68 (24%) due to breast cancer (Table 1). Higher CD163+ TAM and CD68+ TAM counts were both associated with a worse outcome (*p* ≤ 0.023) (Table 2, Supplementary Figure S2), but only the CD163+ TAM count remained significant in the multivariate survival analyses including the standard prognostic factors (*p* < 0.001) (Table 3).

### 3.1. Prognostic Value of TAMs in HER2+ Breast Cancer

**Figure 1:**
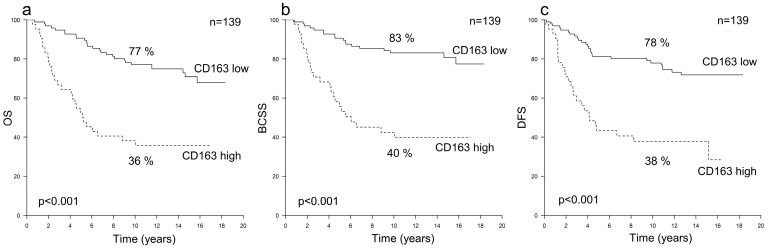
Survival curves illustrating the 10-year survival rates according to the numbers of CD163+ TAMs among all HER2+ cases; overall survival (OS) (**a**), breast cancer-specific survival (BCSS) (**b**) and disease-free survival (DFS) (**c**).

**Figure 2:**
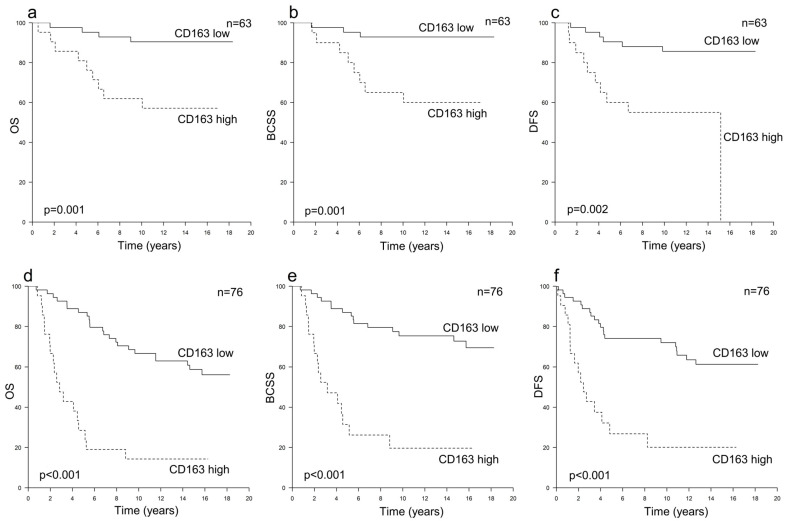
Survival curves according to the numbers of CD163+ TAMs and the use of adjuvant trastuzumab. Panels (**a**–**c**) show overall survival (OS) (**a**), breast cancer-specific survival (BCSS) (**b**) and disease-free survival (DFS) (**c**) among HER2+ patients treated with adjuvant trastuzumab, and panels (**d**–**f**) show OS (**d**), BCSS (**e**) and DFS (**f**) among HER2+ patients that did not receive trastuzumab.

Among the HER2+ cases (*n* = 139), a higher CD163+ TAM count was associated with an inferior outcome across all the investigated survival endpoints (*p* < 0.001). The hazard ratios for every 10-unit increase in CD163+ TAM counts were 1.67 (95% CI 1.37–2.02) for OS, 1.79 (95% CI 1.43–2.23) for BCSS and 1.55 (95% CI 1.28–1.89) for DFS (Table 2). The 10-year OS, BCSS and DFS survival rates among patients with high or low CD163+ TAM counts were 36% vs. 77%, 40% vs. 83% and 38% vs. 78%, respectively (Figure 1). Furthermore, a high CD163+ TAM count was associated with inferior survival irrespective of whether adjuvant trastuzumab was administered (Figure 2) and remained an independent risk factor for poor survival in the multivariate analyses (Table 3). A higher CD68+ TAM count was only statistically significantly associated with a worse BCSS (*p* = 0.041) but did not have any independent prognostic value (Table 2 and Table 3).

### 3.2. Prognostic Value of TAMs in HER2+ Breast Cancer According to Hormone Receptor Status

**Figure 3:**
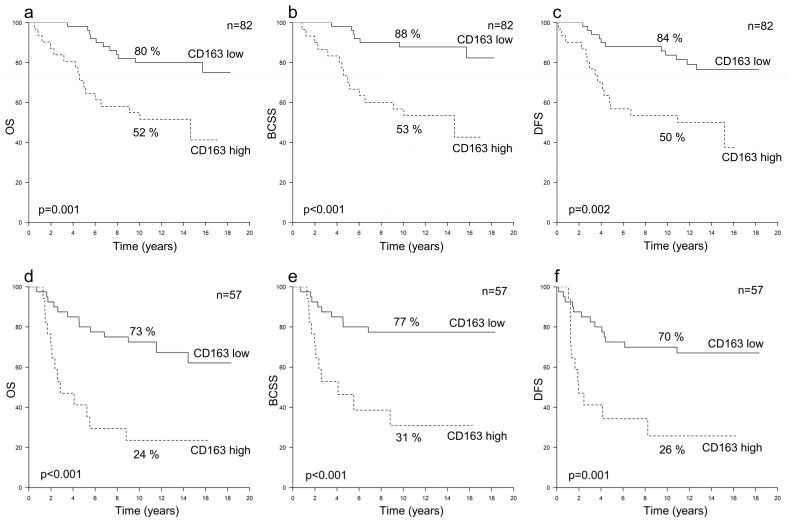
Survival curves illustrating the 10-year survival rates according to the numbers of CD163+ TAMs. Panels (**a**–**c**) show overall survival (OS) (**a**), breast cancer-specific survival (BCSS) (**b**) and disease-free survival (DFS) (**c**) in HER2+/HR+ breast cancer, and panels d–f show OS (**d**), BCSS (**e**) and DFS (**f**) in HER2+/HR− breast cancer.

In both the HER2+/HR+ (*n* = 82) and HER2+/HR− (*n* = 57) subgroups, a high CD163+ TAM count was an independent risk factor for poor OS, BCSS and DFS (*p* ≤ 0.012), and accordingly, the 10-year survival rates were inferior among the patients with high numbers of CD163+ TAMs (*p* ≤ 0.002) (Table 2 and Table 3, Figure 3). In both subgroups, a high CD163+ TAM count was associated with a poor prognosis regardless of the trastuzumab treatment (data not shown). The CD68+ TAM count was not associated with survival in either subgroup (Table 2 and Table 3).

### 3.3. Prognostic Value of TAMs in HER2− Breast Cancer

Neither CD163+ nor CD68+ TAM counts were significantly associated with survival in the HER2-subgroup (*n* = 139) or in the HER2−/HR+ (*n* = 122) or HER2−/HR− (*n* = 17) subgroups (Table 2).

## 4. Discussion

In this study investigating 278 early breast cancer cases with a long follow-up period (median 13.1 years), a high CD163+ TAM count represented a strong independent factor for a poor prognosis. In HER2+ breast cancer, the prognostic effect was evident in both HER2+/HR+ and HER2+/HR− subgroups and was apparent regardless of the administration of adjuvant trastuzumab, whereas among the HER2− cases, the CD163+ TAM count was not significantly associated with survival.

The main finding emerging from this study was that in HER2+ breast cancer, a high CD163+ TAM count was strongly associated with an inferior outcome; it remained a strong independent prognostic factor across all the investigated survival endpoints and regardless of the hormone receptor status, while there were no significant associations with survival in the HER2− cohort. Considering that the numbers of CD163+ TAMs were similar in HER2+ and HER2− tumors, the pro-tumoral functions of CD163+ TAMs can be hypothesized to be especially crucial for the progression of HER2+ breast cancer. Indeed, immunological factors are currently being intensely investigated in this subtype [11,32,33]. Interestingly, the risk factors for HER2+ breast cancer have remained somewhat unclear, but it can be speculated that the inflammatory state may be especially important in the development of HER2+ breast cancer and that macrophages could be important players in this setting. For example, You et al. have reported that in EGFR-positive and HER2-positive basal-like breast cancer, HER2 overexpression leads to significant increases in the secretion of several cytokines, especially Chemokine (C-C motif) ligand 2 (CCL2), which further lead to increased TAM recruitment and production of pro-inflammatory cytokines from M2-polarized TAMs, activating tumorigenesis [34]. In addition, TAMs have been associated with increased angiogenesis in HER2+ breast cancer, and MMP11, a protein found in macrophages, has been found to play a pro-tumoral role in HER2+ breast cancer by enhancing the migration of cancer cells and recruiting monocytes [35]. With respect to the present study, it should be noted that the HER2+ tumors presented with more aggressive features than the HER2− cases, which may have impacted the results, but also reflects the inherent differences between aggressive HER2+ breast cancer and the more indolent luminal cases [36,37], since the majority of the HER2− cases were HR+. Also, further studies will be needed to clarify the mechanisms causing the increased number of CD163+ TAMs. As TAMs are a plastic and diverse cell population, the increased number of CD163+ TAMs may be due to either recruitment or expansion of CD163+ TAMs or because of a shift from the M1-like TAMs towards the M2 direction [38,39].

The prognostic effect of the CD163+ TAM count in HER2+ breast cancer was evident regardless of the administration of adjuvant trastuzumab. This suggests that the pro-tumoral functions of CD163+ TAMs in HER2+ breast cancer are not obstructed by trastuzumab or that CD163+ TAMs might even suppress its actions, even though patients with a high CD163+ TAM count also benefitted from the adjuvant trastuzumab therapy. Indeed, it has been suggested that TAM polarization towards the M2 direction and their recruitment into the TME may impair the activity of anti-HER2 treatments [11,24]. Furthermore, as M1 TAMs have been reported to be associated with improved survival in HER2+ breast cancer [20,22], combining therapies targeting the immunosuppressive microenvironment, e.g., macrophage polarization towards the M1 phenotype, with anti-HER2 treatments could be a beneficial treatment strategy in HER2+ breast cancer. Trastuzumab resistance is a major problem, especially in metastatic HER2+ breast cancer [17,18], and treatments targeting macrophage polarization could be one potential approach to overcoming this obstacle. Indeed, there are ongoing phase 1–2 trials investigating treatments combining anti-HER2 agents with some forms of TAM-directed therapy in HER2+ carcinomas, e.g., HER2-targeted immune-stimulating antibody conjugate locally activating the innate immune system [40] and anti-HER2 CAR macrophage therapy [41]. Nevertheless, there is also evidence suggesting that the efficacy of trastuzumab may be affected more by interactions between different types of immune cells, e.g., the ratios of TILs and TAMs, rather than by the numbers of a single immune cell type on its own [42,43], which highlights the complexity of the immune responses present in the TME.

The prognostic effects of CD68+ TAMs seemed to be somewhat smaller than that of CD163+ TAMs, most probably due to the heterogeneity of the cells expressing the CD68 receptor [9,12]. Our results indicate that CD163+ TAMs appear to represent a more appropriate prognostic biomarker in clinical practice, a finding in line with previous studies [15,44]. The assessment of TAMs has not yet been standardized; along with the different antibodies and counting methods which have been utilized, the determination of the cut-off points for low and high TAM counts have varied in the published studies. Therefore, we decided to investigate the prognostic value of TAM counts as continuous variables to avoid the possible bias inherent in adopting forced cut-off points. Thus, the TAM counts were graded as “low” or “high” only in the multivariate analyses and for plotting the survival curves. When evaluating the numbers of TAMs, we used the hot spot method, as this is a commonly utilized procedure even though it may slightly overestimate the prognostic impact of TAMs [45]. Taken together, while there are several studies which have addressed the prognostic value of TAMs, there are still some issues which need to be clarified before implementing TAM immunohistochemistry into clinical practice.

One of the main strengths of this study is its hospital-based HER2+ patient cohort; it included all operable non-metastatic HER2+ breast cancer cases operated upon in Kuopio University hospital between 2001 and 2008 and thus it represents the real-world HER2+ breast cancer population very well. However, as the HER2− cohort was assembled by matching the HER2+ cohort by age and operation time, it does not represent the whole unselected HER2- patient population. Other strengths of this study include its access to comprehensive patient data as well as the long follow-up time which enabled us to investigate the prognostic value of the TAM counts in different breast cancer subtypes and across several survival endpoints, i.e., OS, BCSS and DFS. One unique feature of this patient cohort is that approximately half of the patients were treated before the trastuzumab era, i.e., adjuvant trastuzumab therapy only became included in the national guidelines in 2005, enabling us to investigate the role of TAMs separately among patients that had received this therapy and among those that had not.

## 5. Conclusions

In this study using real-world patient data, a high M2 (CD163+) TAM count represented a strong independent prognostic factor for an inferior outcome in HER2+ breast cancer; its prognostic value was evident across all the investigated survival endpoints and regardless of the administration of adjuvant trastuzumab and hormone receptor status. These results highlight the important role of M2 TAMs in HER2+ breast cancer and suggest that M2 TAMs could be one of the key factors in the TME enhancing the development and progression of HER2+ breast cancer. In the future, combination treatments including therapies targeting the immunosuppressive TME, such as the polarization of macrophages from pro-tumoral M2 TAMs to anti-tumoral M1 TAMs, could represent a promising approach, especially in HER2+ breast cancer.

## Figures and Tables

**Table 1:** Clinicopathological parameters and the distribution of TAMs in breast cancer subgroups stratified by HER2 status and hormone receptor status.

	All Patients *n* = 278	All HER2+ *n* = 139	HER2+/HR+ *n* = 82	HER2+/HR− *n* = 57	All HER2− *n* = 139
**Age, median (range)**	58.6 (32–86)	58.7 (32–86)	59.3 (32–86)	58.2 (40–82)	58.6 (33–84)
**Adjuvant treatment, *n* (%)**					
Chemotherapy	204 (73)	110 (79)	62 (76)	48 (84)	94 (68)
Radiation therapy	248 (89)	124 (89)	72 (88)	52 (91)	124 (89)
Hormonal therapy	174 (63)	73 (53)	71 (87)	2 (4)	101 (73)
Trastuzumab	63 (45 *)	63 (45)	36 (44)	27 (47)	0 (0)
**Tumor classification, *n* (%)**					
pT1	156 (56)	67 (48)	45 (55)	22 (39)	89 (64)
pT2	99 (36)	60 (43)	32 (39)	28 (49)	39 (28)
pT3	10 (4)	4 (3)	2 (2)	2 (4)	6 (4)
pT4	13 (5)	8 (6)	3 (4)	5 (9)	5 (4)
**Nodal classification, *n* (%)**					
pN0	103 (37)	47 (34)	34 (41)	13 (23)	56 (40)
pN1	121 (44)	55 (40)	29 (35)	26 (46)	66 (48)
pN2	35 (13)	22 (16)	12 (15)	10 (18)	13 (9)
pN3	19 (7)	15 (11)	7 (9)	8 (14)	4 (3)
**Histological grade, *n* (%)**					
1	23 (8)	4 (3)	4 (5)	0 (0)	19 (14)
2	123 (44)	47 (34)	33 (40)	14 (25)	76 (55)
3	132 (47)	88 (63)	45 (55)	43 (75)	44 (32)
**Histological type, *n* (%)**					
Ductal	228 (82)	119 (86)	72 (88)	47 (82)	109 (78)
Lobular	27 (10)	9 (6)	4 (5)	5 (9)	18 (13)
Other	23 (8)	11 (8)	6 (7)	5 (9)	12 (9)
**HR+, *n* (%)**	139 (50)	82 (59)	82 (100)	0 (0)	122 (88)
**CD163+ TAMs, median (range)**	26 (5–65)	28 (8–64)	26 (9–64)	30 (8–60)	24 (5–65)
**CD68+ TAMs, median (range)**	32 (7–73)	38.5 (13–73)	35.5 (13–60)	41.5 (13–73)	29 (7–60)
**Any relapse, *n* (%)**	91 (33)	53 (38)	28 (34)	25 (44)	38 (27)
**Distant metastases, *n* (%)**	73 (26)	45 (32)	23 (28)	22 (39)	28 (20)
**Death, *n* (%)**	98 (35)	55 (40)	28 (34)	27 (47)	43 (31)
**Breast cancer death, *n* (%)**	68 (24)	43 (31)	23 (28)	20 (35)	25 (18)

HR+, hormone receptor-positive; HR−, hormone receptor-negative, TAMs; tumor-associated macrophages, * of HER2+ cases.

**Table 2:** The number of TAMs as a prognostic marker for OS, BCSS and DFS: the hazard ratios for 10-unit increases in CD163+ and CD68+ TAM counts are presented.

		OS HR (95% CI), *p*	BCSS HR (95% CI), *p*	DFS HR (95% CI), *p*
**All** (*n* = 278)	CD163+ TAMs	1.55 (1.33–1.80), <0.001 *	1.64 (1.37–1.96), <0.001 *	1.45 (1.24–1.70), <0.001 *
	CD68+ TAMs	1.20 (1.03–1.40), 0.022 *	1.30 (1.08–1.57), 0.005 *	1.20 (1.03–1.40), 0.023 *
**All HER2+** (*n* = 139)	CD163+ TAMs	1.67 (1.37–2.02), <0.001 *	1.79 (1.43–2.23), <0.001 *	1.55 (1.28–1.89), <0.001 *
	CD68+ TAMs	1.23 (1.00–1.51), 0.057	1.28 (1.01–1.63), 0.041 *	1.12 (0.91–1.38), 0.296
**HER2+/HR+** (*n* = 82)	CD163+ TAMs	1.64 (1.26–2.14), <0.001 *	1.85 (1.37–2.51), <0.001 *	1.67 (1.27–2.19), <0.001 *
	CD68+ TAMs	1.14 (0.85–1.53), 0.367	1.29 (0.93–1.78), 0.133	1.18 (0.89–1.58), 0.255
**HER2+/HR−** (*n* = 57)	CD163+ TAMs	1.80 (1.33–2.45), <0.001 *	1.79 (1.28–2.53), <0.001 *	1.48 (1.09–2.02), 0.012 *
	CD68+ TAMs	1.24 (0.91–1.69), 0.165	1.22 (0.86–1.75), 0.271	1.00 (0.73–1.36), 0.987
**All HER2−** (*n* = 139)	CD163+ TAMs	1.28 (1.00–1.65), 0.052	1.22 (0.88–1.70), 0.235	1.22 (0.94–1.58), 0.139
	CD68+ TAMs	1.06 (0.83–1.36), 0.642	1.15 (0.84–1.58), 0.372	1.23 (0.96–1.58), 0.102
**HER2-/HR+** (*n* = 122)	CD163+ TAMs	1.25 (0.92–1.69), 0.150	1.17 (0.78–1.74), 0.453	1.27 (0.94–1.72), 0.117
	CD68+ TAMs	0.99 (0.74–1.33), 0.951	1.02 (0.70–1.48), 0.926	1.25 (0.93–1.66), 0.138
**HER2-/HR−** (*n* = 17)	CD163+ TAMs	1.30 (0.69–2.44), 0.418	1.16 (0.51–2.63), 0.721	1.16 (0.51–2.63), 0.721
	CD68+ TAMs	1.17 (0.62–2.20), 0.621	1.75 (0.66–4.63), 0.262	1.75 (0.66–4.63), 0.262

TAMs, tumor-associated macrophages; OS, overall survival; BCSS, breast cancer-specific survival; DFS, disease-free survival; HR+, hormone receptor-positive; HR−, hormone receptor-negative; * *p* < 0.05.

**Table 3:** Cox multivariate analyses for OS, BCSS and DFS presenting the data for CD163+ and CD68+ TAM counts and standard prognostic factors.

	OS			BCSS			DFS		
	*p*-Value	HR	95% CI	*p*-Value	HR	95% CI	*p*-Value	HR	95% CI
**All patients (*n* = 278)**									
CD163+ TAMs	<0.001 *	2.71	1.77–4.14	<0.001 *	3.36	2.04–5.55	<0.001 *	2.37	1.53–3.66
CD68+ TAMs	0.469	1.19	0.74–1.91	0.264	1.35	0.80–2.30	0.476	1.19	0.74–1.91
Tumor size	0.002 *	1.98	1.27–3.08	0.001 *	2.50	1.42–4.39	0.070	1.52	0.97–2.37
Nodal status	0.015 *	1.81	1.12–2.94	0.001 *	3.09	1.56–6.13	0.001 *	2.34	1.40–3.93
Grade	0.711	1.09	0.69–1.73	0.096	1.62	0.92–2.86	0.045 *	1.64	1.01–2.65
Hormone receptor- status	0.254	0.76	0.48–1.21	0.701	0.90	0.53–1.54	0.928	0.98	0.61–1.58
HER2 status	0.300	1.26	0.82–1.94	0.181	1.42	0.85–2.39	0.337	1.24	0.80–1.93
**All HER2+ (*n* = 139)**									
CD163+ TAMs	<0.001 *	4.41	2.51–7.77	<0.001 *	5.67	2.94–10.95	<0.001 *	4.13	2.32–7.36
CD68+ TAMs	0.104	1.77	0.89–3.52	0.089	2.05	0.90–4.70	0.344	1.38	0.71–2.69
Tumor size	0.201	1.52	0.80–2.89	0.433	1.37	0.63–2.98	0.665	1.15	0.61–2.19
Nodal status	0.072	1.86	0.95–3.64	0.005 *	4.09	1.54–10.89	<0.001 *	4.07	1.79–9.25
Grade	0.402	1.34	0.68–2.67	0.158	1.82	0.79–4.15	0.079	1.88	0.93–3.82
Hormone receptor- status	0.091	0.61	0.35–1.08	0.242	0.68	0.36–1.30	0.126	0.64	0.35–1.14
Adjuvant trastuzumab	<0.001 *	0.23	0.12–0.45	<0.001 *	0.27	0.13–0.57	0.001*	0.36	0.20–0.67
**HER2+/HR+ (*n* = 82)**									
CD163+ TAMs	0.003 *	3.62	1.55–8.45	<0.001 *	5.44	2.05–14.44	0.002 *	3.71	1.60–8.62
CD68+ TAMs	0.592	1.28	0.52–3.20	0.485	1.46	0.51–4.22	0.573	1.29	0.53–3.11
Tumor size	0.425	1.51	0.55–4.11	0.950	1.04	0.32–3.33	0.639	0.79	0.30–2.09
Nodal status	0.238	1.85	0.67–5.11	0.055	3.79	0.97–14.77	0.034 *	3.20	1.09–9.36
Grade	0.210	1.87	0.70–4.95	0.112	2.56	0.80–8.15	0.045 *	2.73	1.02–7.28
Adjuvant trastuzumab	0.007 *	0.27	0.11–0.71	0.015 *	0.28	0.10–0.78	0.013 *	0.32	0.13–0.79
**HER2+/HR− (*n* = 57)**									
CD163+ TAMs	<0.001 *	4.20	1.81–9.74	<0.001 *	5.46	2.02–14.79	<0.001 *	4.98	1.95–12.75
CD68+ TAMs	0.331	1.58	0.63–4.00	0.168	2.46	0.68–8.84	0.216	1.93	0.68–5.47
Tumor size	0.504	1.35	0.56–3.29	0.633	1.33	0.41–4.35	0.475	1.41	0.55–3.59
Nodal status	0.107	2.25	0.84–6.04	0.018 *	6.50	1.37–30.82	0.004 *	9.75	2.07–45.94
Grade	0.677	0.81	0.30–2.17	0.697	1.28	0.37–4.38	0.259	1.88	0.63–5.60
Adjuvant trastuzumab	0.001 *	0.20	0.08–0.53	0.018 *	0.26	0.09–0.80	0.037 *	0.39	0.16–0.95

OS, overall survival; BCSS, breast cancer-specific survival; DFS, disease-free survival; TAMs, tumor-associated macrophages; HR+, hormone receptor-positive; HR−, hormone receptor-negative; * *p* < 0.05.

## Data Availability

The data are not publicly available due to privacy and ethical restrictions. The data that support the findings of this study may be made available from the corresponding author on reasonable request and with the required permissions.

## Supplementary Materials

The following supporting information can be downloaded at: https://www.mdpi.com/article/10.3390/cancers16030634/s1, Figure S1: Examples of immunohistochemical staining of the tumor-associated macrophages (TAMs). The stained (brown) cells that appear morphologically as macrophages indicate CD163+ TAMs (panel a) and CD68+ TAMs (panel b); Figure S2: Survival curves presenting 10-year survival rates according to the numbers of TAMs in the whole patient cohort. Panels a-c show overall survival (OS) (a), breast cancer-specific survival (BCSS) (b) and disease free-survival (DFS) (c) according to the numbers of CD163+ TAMs, and panels d–f show OS (d), BCSS (e) and DFS (f) according to the numbers of CD68+ TAMs; Table S1: The numbers of OS, BCSS and DFS events in the investigated breast cancer subgroups.
