# Supplementary material for: High Numbers of CD163+ Tumor-Associated Macrophages Predict Poor Prognosis in HER2+ Breast Cancer
Source: Cancers (Basel). 2024 Feb 1;16(3):634. doi: 10.3390/cancers16030634 (PMC10854814; doi:10.3390/cancers16030634)
Supplement: Supplementary file 1 [file cancers-16-00634-s001.zip › suppltable.pdf]

**Supplementary Table S1.** The numbers of OS, BCSS and DFS events in the investigated breast cancer subgroups

| | Number of patients | OS event* n (%) | BCSS event** n (%) | DFS event*** n (%) | Median follow-up time years (range) |
|---|---|---|---|---|---|
| **All** | 278 | 98 (35) | 68 (24) | 91 (33) | 13.1 (0.4-18.8) |
| CD163+ high | 75 | 41 (55) | 33 (44) | 37 (49) | |
| CD163+ low | 201 | 56 (28) | 34 (17) | 53 (26) | |
| CD68+ high | 60 | 26 (43) | 22 (37) | 27 (45) | |
| CD68+ low | 210 | 67 (32) | 43 (20) | 61 (29) | |
| **HER2+** | 139 | 55 (40) | 43 (31) | 53 (38) | 12.7 (0.4-18.3) |
| CD163+ high | 42 | 27 (64) | 24 (57) | 26 (62) | |
| CD163+ low | 96 | 27 (28) | 18 (19) | 26 (27) | |
| CD68+ high | 90 | 41 (46) | 32 (36) | 36 (40) | |
| CD68+ low | 46 | 11 (24) | 8 (17) | 14 (30) | |
| **HER2+/HR+** | 82 | 28 (34) | 23 (28) | 28 (34) | 13.0 (0.4-18.3) |
| CD163+ high | 31 | 16 (52) | 15 (48) | 16 (52) | |
| CD163+ low | 50 | 11 (22) | 7 (14) | 11 (22) | |
| CD68+ high | 47 | 19 (40) | 16 (34) | 18 (38) | |
| CD68+ low | 33 | 7 (21) | 5 (15) | 8 (24) | |
| **HER2+/HR-** | 57 | 27 (47) | 20 (35) | 25 (44) | 11.6 (0.7-18.3) |
| CD163+ high | 17 | 13 (76) | 11 (65) | 12 (71) | |
| CD163+ low | 40 | 14 (35) | 9 (23) | 13 (33) | |
| CD68+ high | 37 | 19 (51) | 15 (41) | 17 (46) | |
| CD68+ low | 19 | 7 (37) | 4 (21) | 7 (37) | |
| **HER2-** | 139 | 43 (31) | 25 (18) | 38 (27) | 13.5 (0.9-18.8) |
| CD163+ high | 37 | 16 (43) | 10 (27) | 13 (35) | |
| CD163+ low | 101 | 27 (27) | 15 (15) | 25 (25) | |
| CD68+ high | 19 | 8 (42) | 7 (37) | 10 (53) | |
| CD68+ low | 115 | 33 (29) | 18 (16) | 28 (24) | |
| **HER2-/HR+** | 121 | 37 (31) | 21 (17) | 34 (28) | 13.6 (2.1-18.8) |
| CD163+ high | 27 | 11 (41) | 7 (26) | 10 (37) | |
| CD163+ low | 94 | 26 (28) | 14 (15) | 24 (26) | |
| CD68+ high | 42 | 9 (21) | 5 (12) | 13 (31) | |
| CD68+ low | 75 | 26 (35) | 16 (21) | 21 (28) | |
| **HER2-/HR-** | 17 | 6 (35) | 4 (24) | 4 (24) | 12.9 (0.9-18.4) |
| CD163+ high | 10 | 5 (50) | 3 (30) | 3 (30) | |
| CD163+ low | 7 | 1 (14) | 1 (14) | 1 (14) | |
| CD68+ high | 7 | 3 (43) | 3 (43) | 3 (43) | |
| CD68+ low | 10 | 3 (30) | 1 (10) | 1 (10) | |

OS, overall survival; BCSS, breast cancer-specific survival; DFS disease-free survival; HR+, hormone receptor positive; HR-, hormone receptor negative
*OS event=death; **BCSS event=breast cancer death; ***DFS event=disease recurrence
