# Supplementary figures and images for: High Numbers of CD163+ Tumor-Associated Macrophages Predict Poor Prognosis in HER2+ Breast Cancer
Source: Cancers (Basel). 2024 Feb 1;16(3):634. doi: 10.3390/cancers16030634 (PMC10854814; doi:10.3390/cancers16030634)

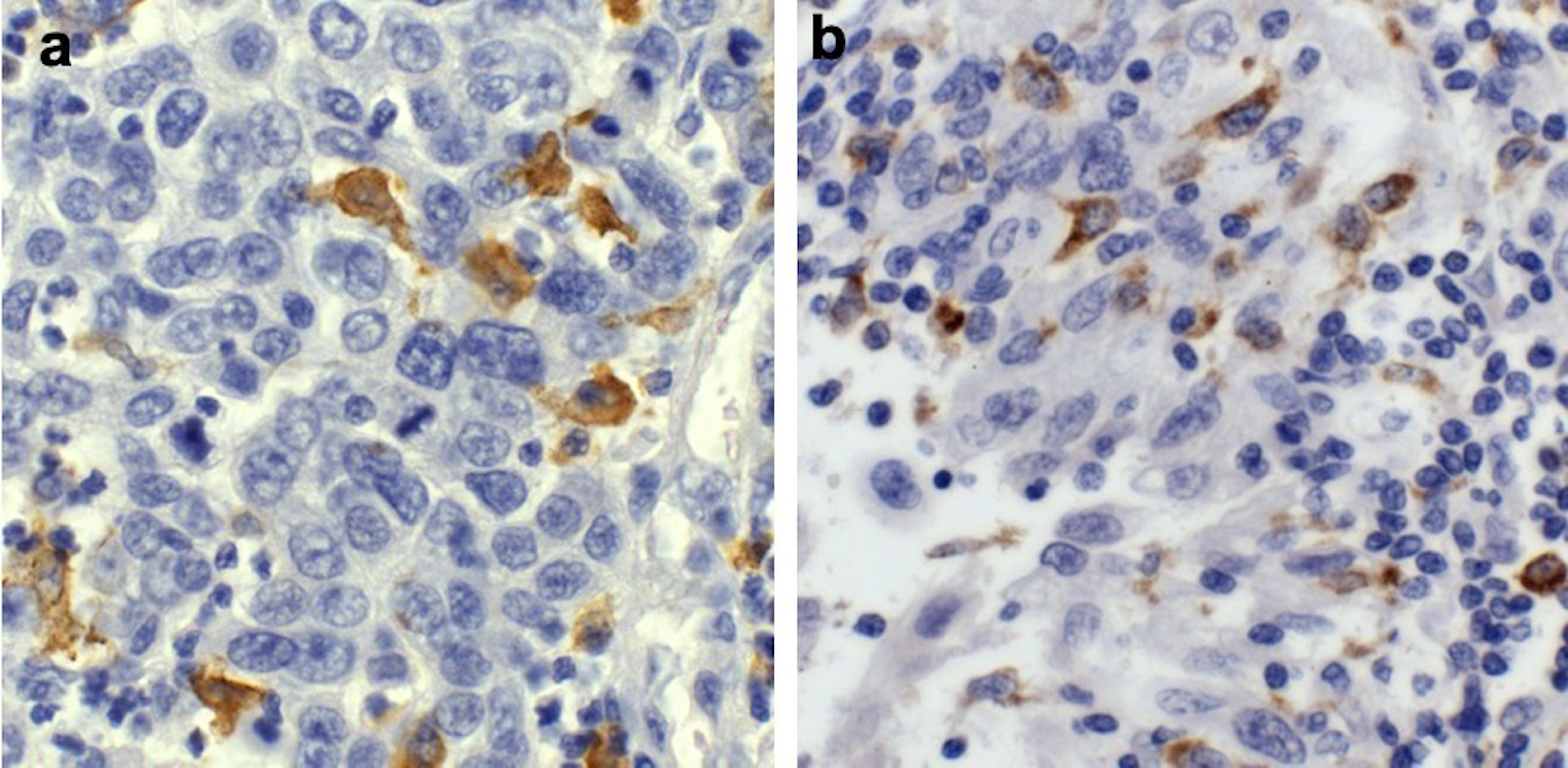

Supplement: Supplementary file 1 [file cancers-16-00634-s001.zip › suppl_Fig1.tiff]

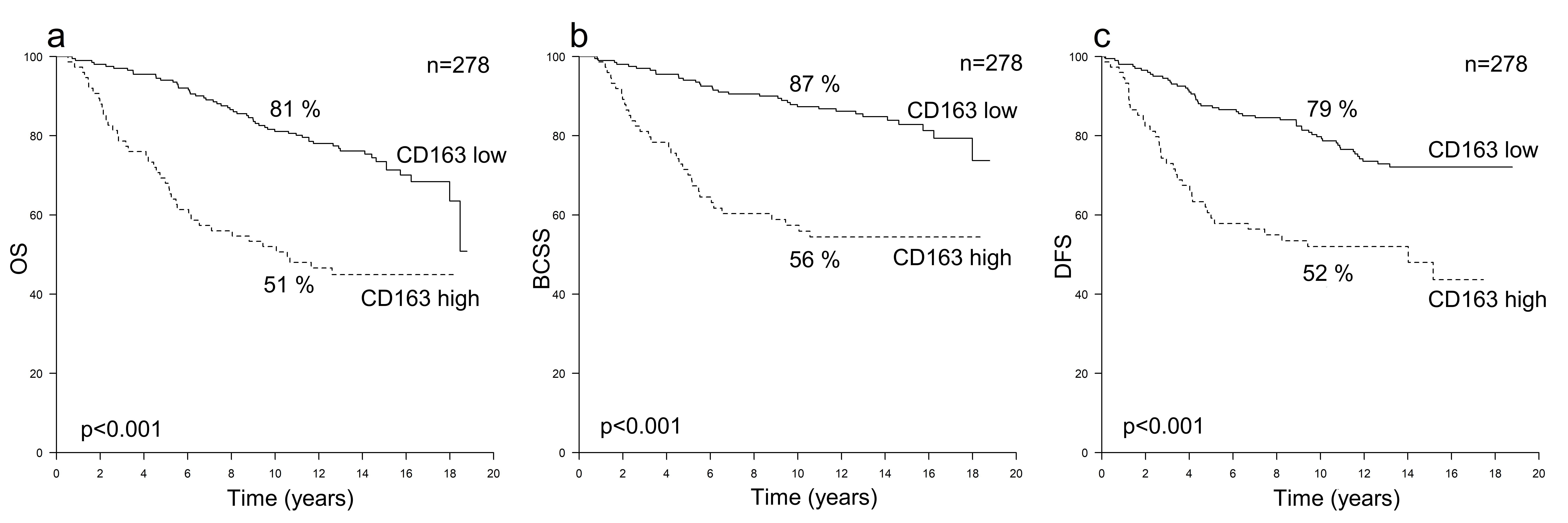

Supplement: Supplementary file 1 [file cancers-16-00634-s001.zip › suppl_Fig2_abc_rev.tiff]

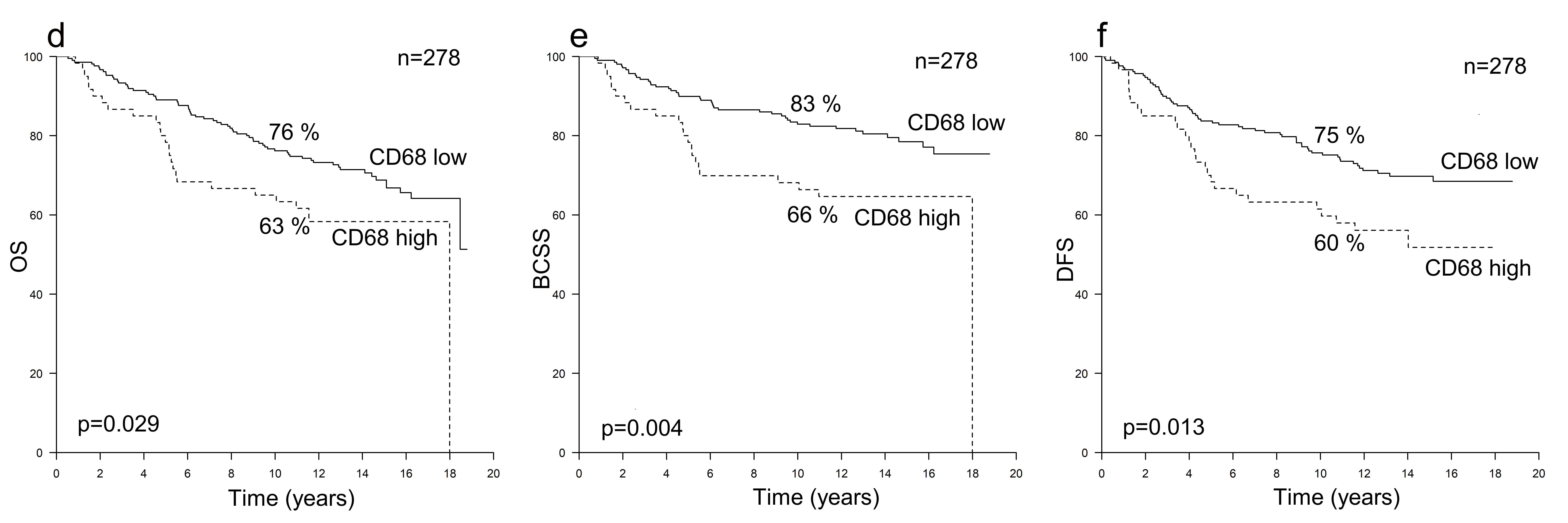

Supplement: Supplementary file 1 [file cancers-16-00634-s001.zip › suppl_Fig2_def_rev.tiff]
